# Applications of magnetic resonance-guided laser interstitial thermal therapy in disconnective epilepsy surgery

**DOI:** 10.3389/fneur.2024.1484263

**Published:** 2024-12-02

**Authors:** Fidelia Gaba, Jasmine L. Hect, Taylor J. Abel

**Affiliations:** ^1^Department of Neurological Surgery, University of Pittsburgh School of Medicine, Pittsburgh, PA, United States; ^2^Department of Bioengineering, University of Pittsburgh Medical Center, Pittsburgh, PA, United States

**Keywords:** epilepsy, corpus callosotomy, hemispherotomy, tractography, pediatric neurosurgery

## Abstract

Minimally invasive surgical techniques, such as MR-guided laser interstitial thermal therapy (LITT), have emerged as promising alternatives to open disconnective surgeries in drug-resistant epilepsy (DRE). This review synthesizes current literature on the application of LITT for corpus callosal disconnection and functional hemispheric disconnection. Studies highlight LITT's effectiveness for achieving seizure control and functional outcomes, often with reduced complications compared to traditional open procedures. Challenges include technical limitations to achieving total disconnection and adequate assessment of disconnection postoperatively. The literature is largely composed of observational studies and there is a need for rigorous, multi-center trials to establish robust guidelines and improve generalizability in clinical practice. There is also a need for a more robust exploration of how patient-specific factors contribute to response or nonresponse to intervention.

## 1 Introduction

Up to 40% of patients with epilepsy have drug resistant epilepsy (DRE) and should be referred for surgical evaluation (1, 2). However, due to a myriad of barriers, epilepsy surgery continues to be underutilized (3). Importantly, one source of underutilization is the stigma of epilepsy surgery and patient or caregiver resistance to open cranial epilepsy surgeries and associated risks (1). Uncontrolled epilepsy is associated with decreased quality of life, neurodevelopmental regression, physical injury, and death (4–7). Meanwhile, surgical intervention for DRE is associated with significant improvements in quality of life (8) and cognitive development (9–11).

To address the morbidity of epilepsy surgery, several minimally invasive techniques have been developed. Endoscopic approaches are less invasive than open surgery, but require craniotomy and interhemispheric dissection with the associated inherent risks (12, 13). Gamma knife radiosurgery is the least invasive option, but it comes with the potential for radiation necrosis and is not immediately effective (14, 15). Finally, Laser Interstitial Thermal Therapy (LITT) eliminates the need for craniotomy (16) and avoids radiation-related complications (e.g., radiation necrosis and mutagenic risks), which is a significant advantage for use in pediatric populations (17, 18).

**Figure 1:**
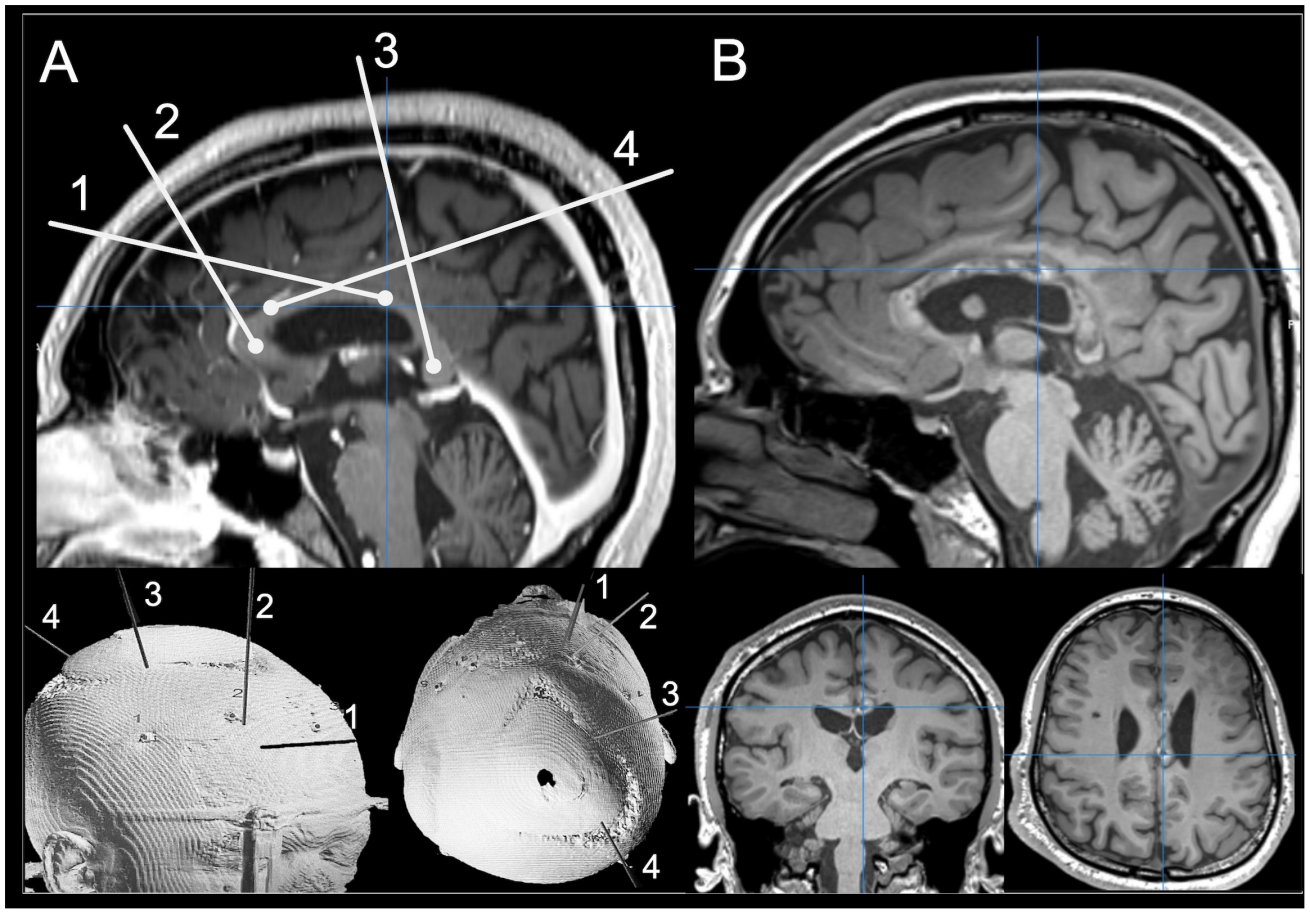
**(A)** Preoperative planning photo for a complete CCA using mid-sagittal T1-weighted MRI images and 3D rendering for the patient's skull. **(B)** Postoperative T1-weighted MRI images scans showing ablated tissue on mid-sagittal T1-weighted MRI images with coronal and axial views.

LITT is a minimally invasive neurosurgical technique used to treat a variety of brain pathologies via targeted thermal ablation (19). It was first described for treatment of seizure foci in 2012 and is now used for various focal epilepsy etiologies (20). During the procedure a laser probe is placed along a planned trajectory using stereotactic technique (Figure 1). Light energy emitted by the laser converts to heat, inducing coagulation necrosis to ablate the tissue (19). Compared to open surgical techniques, LITT is associated with a more favorable complication profile (21–37), shorter hospital stays, and non-inferior seizure outcomes in epilepsy surgery (22–33). However, many studies, including recent prospective observational non-inferiority analyses, suggest that LITT is less effective than traditional epilepsy surgery (21, 38–41). Therefore, the role of LITT relative to traditional approaches is an open debate and remains to be fully evaluated. However, when used in certain disconnective contexts—such as corpus callosotomy—LITT seems to have a similar effectiveness to open microsurgical corpus callosotomy based on recent systematic reviews (42).

For patients with DRE who experience drop seizures, when resection of an epileptogenic focus is not possible vagus nerve stimulation (VNS) or disconnective surgery are alternative treatment options. VNS is widely used globally as an adjunctive therapy to reduce seizure frequency in both adults and children (43). Its safety and effectiveness have been well-established across many epilepsy centers (44–47) and it is associated with significant improvements in health-related quality of life and reduced use of hospital services among DRE patients including those with drop seizures (48, 49). Furthermore, a retrospective single-center study found that 57.8% of VNS patients experienced reduced overall seizure frequency or total seizure freedom at last follow-up (50) and a 2022 meta-analysis of 5,223 pediatric and adult patients demonstrated increased effectiveness of VNS over time, with responder rates improving from 42.1% at 3 month to 50.8% at 60 months (51).

Despite VNS being generally well-tolerated and effective, patients are significantly more likely to achieve a >50% reduction in overall seizure frequency with CC than with VNS (88.6 vs. 52.5%). This trend is particularly pronounced with drop seizures, the most injurious seizure type, with 58.0% of callosotomy patients achieving drop seizure freedom compared to only 21.1% of VNS patients. In addition, adverse events (hoarseness and voice changes) were more common with VNS though they are typically milder than those associated with corpus callosotomy (e.g., disconnection syndrome) (52).

Disconnective techniques such as corpus callosotomy and functional hemispherotomy control seizures by surgically interrupting fibers in an epileptic network (53). In the case of callosotomy, this disconnection slows the rapid spread of ictal activity between the two cerebral hemispheres that leads to drop seizures—characterized by sudden loss of motor tone, leading to falls and traumatic injuries (54). In the case of hemispherotomy, disconnection completely disconnects a hemispheric seizure focus. LITT has emerged as a minimally invasive alternative or adjunct to these open procedures. However, much like LITT as a whole relative to open surgery, the contemporary clinical applications and limitations of LITT in disconnective epilepsy surgeries remain unclear. In this review we explore the use of LITT for corpus callosum ablation and hemispherotomy.

## 2 Literature search and screening methodology for this narrative review

On September 24, 2024 a database search of PubMed (National Library of Medicine), Embase (Elsevier), and Cochrane Library (Wiley) was performed by F.G. using the following search line:

(epilepsy OR refractory epilepsy OR lennox-gastaut syndrome OR drug resistant epilepsy OR generalized epilepsy OR intractable epilepsy OR seizures OR drop seizures OR drop attacks OR atonic seizures) AND (laser interstitial thermal therapy OR MRgLITT OR LITT OR laser ablation OR laser callosotomy, OR MRI-guided OR stereotactic functional neurosurgery OR laser OR MR-guided) AND (disconnective surgery OR epilepsy surgery OR disconnection OR redisconnection OR reoperation OR hemispherectomy OR hemispherotomy OR anatomic hemispherectomy OR functional hemispherectomy OR reoperative hemispherectomy OR corpus callosotomy OR CC OR callosotomy OR robotic-assisted callosotomy OR corpus callosum)

**Figure 2:**
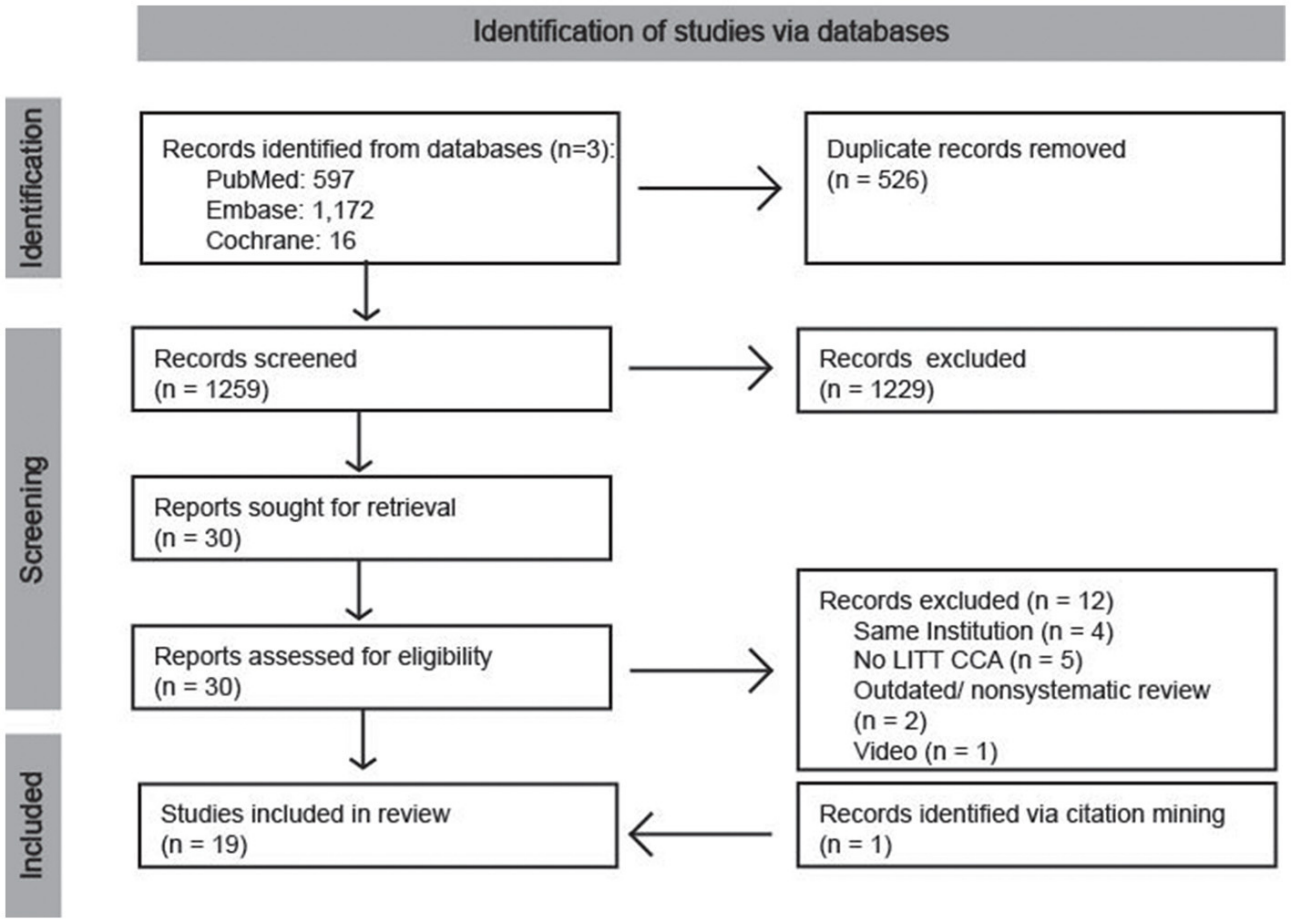
Flow diagram of the LITT search and study selection for this narrative review.

The results of the database searches were downloaded and compiled in excel (version 16.78.3) and a manual screening process was performed. First, records were sorted by their digital object identifier and titles and duplicates were removed. Then, titles were screened, followed by abstracts. Finally, articles were retrieved and assessed for eligibility. One study was identified through citation mining and manually added following the initial search (Figure 2).

Articles that reported on the wrong seizure semiologies (e.g., glioma, tuberous sclerosis, focal epilepsies) or the wrong procedure (e.g., gamma knife, ultrasound, stereoelectroencephalography, vagus nerve stimulation, amygdalohippocampectomy, etc.) were excluded. Nonsystematic reviews and systematic reviews published before 2023 were also excluded. To be eligible for this study articles had to report on human subjects only and include patients with drug resistant epilepsy who underwent LITT for either corpus callosum ablation or hemispherotomy (including functional hemispherotomy and completion of a prior disconnection). Studies included in this review are recorded in Tables 1, 2 for corpus callosum ablation and hemispherotomy, respectively.

**Table 1:** Characteristics of included CCA studies.

**References**	**Study type**	**Investigation dates**	**Patients, *n***	Mean Age, yrs (range^*^)
Aum et al. (54)	Retrospective cohort	2003–2021	103	9.9 (6.2–14.9)
Ball et al. (25)	Case report	ND	1	21.0
Best et al. (26)	Case series	2020–2021	3	19.3 (14–27)
Caruso et al. (23)	Case-control	2005–2018	7	10.6 (ND)
Hect et al. (42)	Systematic review	2016–2023^**^	85	20.8 (1–52)
Ho et al. (27)	Case report	ND	1	30.0
Huang et al. (28)	Case Series	2015–2018	6	22.8 (8–40)
Lehner et al. (30)	Case series	2015–2017	5	28.2 (21–44)
Ordaz et al. (22)	Case series	2019–2021	9	11.4 (5–18)
Palma et. al (32)	Case series	ND	3	12.4 (1–23)
Phillips et al. (60)	Retrospective cohort	1994–2022	36	12(9–17)
Pruitt et al. (33)	Case series	2009–2015	3	ND
Rich et al. (34)	Case series	2014–2018	13	31.2 (20–49)
Tao et al. (36)	Case series	2014–2019	10	33.5 (11–52)
Ung et al. (37)	Case series	ND	2	21.5 (18–25)

^*^Interquartile range.

^**^Articles published between these two dates.

Yrs, years; ND, no data available.

**Table 2:** Seizure outcomes of complete functional hemispherotomy studies.

**References**	**Patients, *n***	**Patient age range (yrs)**	**DS free**	**Longest follow-up (mos)**	**Complications**
**Completion of prior disconnective surgery**					
Candela-Cantó et al. (24)	6	4–18	4/6	29	None
Ravindra et al. (85)	5	1.8–12.9	2/5	30.6	None
**Functional hemispherotomy**					
Chua et al. (86)	1	5	1/1	16	Transient ICP increases and vomiting
Mendoza-Elias et al. (87)	2	11	2/2	9	Small intraventricular/ subarachnoid hemorrhage

Yrs, years; mos, months; DS, drop seizures.

## 3 Applications of LITT to corpus callosotomy

### 3.1 Surgeries performed

First described by Van Wagenen in the 1940s, corpus callosotomy is one of the most common and effective treatments for drop seizures; it also leads to significant reductions in tonic seizures and partial seizures with rapid secondary generalization (55–57). While less effective than callosotomy, many patients undergo VNS first, likely because it seems less invasive (58). Recently, LITT corpus callosum ablation (CCA) emerged a minimally invasive alternative to traditional open corpus callosotomy (23, 31, 35, 59, 60). In a 2024 retrospective cohort study, Phillips et al. reported significantly lower prior VNS (47%) in CCA patients compared to open callosotomy patients (80%) (60). This suggests that the minimally invasive nature of LITT CCA may make patients and their families more amenable to pursuing it as an initial procedure.

Similar seizure outcomes have been reported for CCA and open callosotomy. In their 2023 meta-analysis, Wu et al. reported total seizure freedom rates of 12.38% and freedom from drop seizures of 61.86% in patients with at least 1 year of follow-up after callosotomy (61). Similarly, Hect et al. found overall seizure freedom rates of 18.87% and drop seizure freedom of 46.28% in patients with at least 6 months follow-up after CCA (42). These findings should be considered cautiously as long-term follow-up data is limited, and the timing to achieve seizure freedom varies: some patients experience immediate reduction or freedom from drop seizures, while others respond gradually over weeks to months. Also, some studies report increased seizure burden or new semiologies post-CCA. Finally, as with open callosotomy, CCA's effectiveness in reducing atonic seizures appears to diminish over time. Nevertheless, there may still be long-term improvements in patient quality of life even in light of continued seizure activity: Phillips et al. found that medication burden remained decreased or unchanged in 83% of patients at longest follow-up post-CCA (60).

The most common applications of LITT for corpus callosum ablation has been for complete CCA (27.78%), anterior two-thirds CCA (38.89%), and posterior one-third CCA for completion of a prior partial CCA (22.22%) (42). Upfront complete CCAs were often performed in the setting of prior VNS implantation with insufficient seizure control with rates ranging from 20 to 100% (22, 23, 25, 26, 37, 60) and Hect et al. found that by last follow-up, 62.50% of patients had undergone VNS placement. Taken together with the 2020 meta-analysis by Ye et al., these findings suggest that VNS may enhance seizure control in conjunction with CCA and that some patients may be poor responders to VNS and would benefit from earlier or initial intervention with CCA (62).

Of the 45 patients, reported by Hect et al., with complete anatomical CCA at the most recent follow-up, one-quarter required additional surgery as anterior two-thirds disconnection was insufficient for seizure freedom (42). Numerous studies suggest that single stage complete corpus callosotomy yields better outcomes for drop seizures than anterior two-thirds callosotomy (55, 63–71). However, due to severe functional losses associated with complete callosotomy, partial callosotomy is preferred in patients without significant developmental delays and those with intact language abilities and ambulation (26, 54, 62, 72). It is worth considering the effects that repeat procedures and prolonged seizure burden can have on patient and family quality of life, as well as the long-term impact on development. Unfortunately, not all patients who respond poorly to anterior two-thirds callosotomy/CCA show significant improvement beyond their initial seizure control after follow-up posterior disconnection (54), suggesting that increasing the rate of upfront complete CCA without careful consideration is not advisable. This highlights the need for improved methods to identify patients who are responders and non-responders to anterior CCA to optimize patient outcomes.

### 3.2 Surgical complications

While the rates of seizure freedom are comparable, and complication rates are similar, open corpus callosotomy carries a far less favorable complication profile. This includes all the risks of cranial surgery as well as the risk for complications such as hydrocephalus or persistent CSF leak requiring a shunt, disconnection syndrome, retraction injury to the cingulate gyrus, and vascular injury to the pericallosal arteries (23, 73). Beyond having a more favorable complication profile, CCA has also been associated with lower blood loss and shorter hospital stays (42, 54).

The two most common surgical complications associated with CCA are probe malpositioning and hemorrhage: out of 90 CCA operations, Hect et al. documented six cases of probe malpositioning and five cases of hemorrhages (42). Accurate probe placement is critical for completeness of CCA and effective seizure control (22). Lehner et al. reported misplacing two of three catheters in a case which resulted in incomplete ablation of the corpus callosum and necessitated a follow-up procedure (30). Beyond technical errors, a thin or tortuous corpus callosum can complicate laser targeting (35).

The documented hemorrhages occurred in five patients across five institutions, usually at the probe entry site or along its trajectory. However, Pruitt et al. has described a case where hemorrhage occurred upon removal of a laser probe likely caused by a bent probe that overheated and charred surrounding tissue (33). None of the hemorrhages required surgical intervention or led to permanent neurological deficit. Other documented complications include ablation of a non-target area due to extension of the heat applied from the splenium into the left thalamus, infection, and CSF leak, with no reported patient deaths during or after CCA.

**Figure 3:**
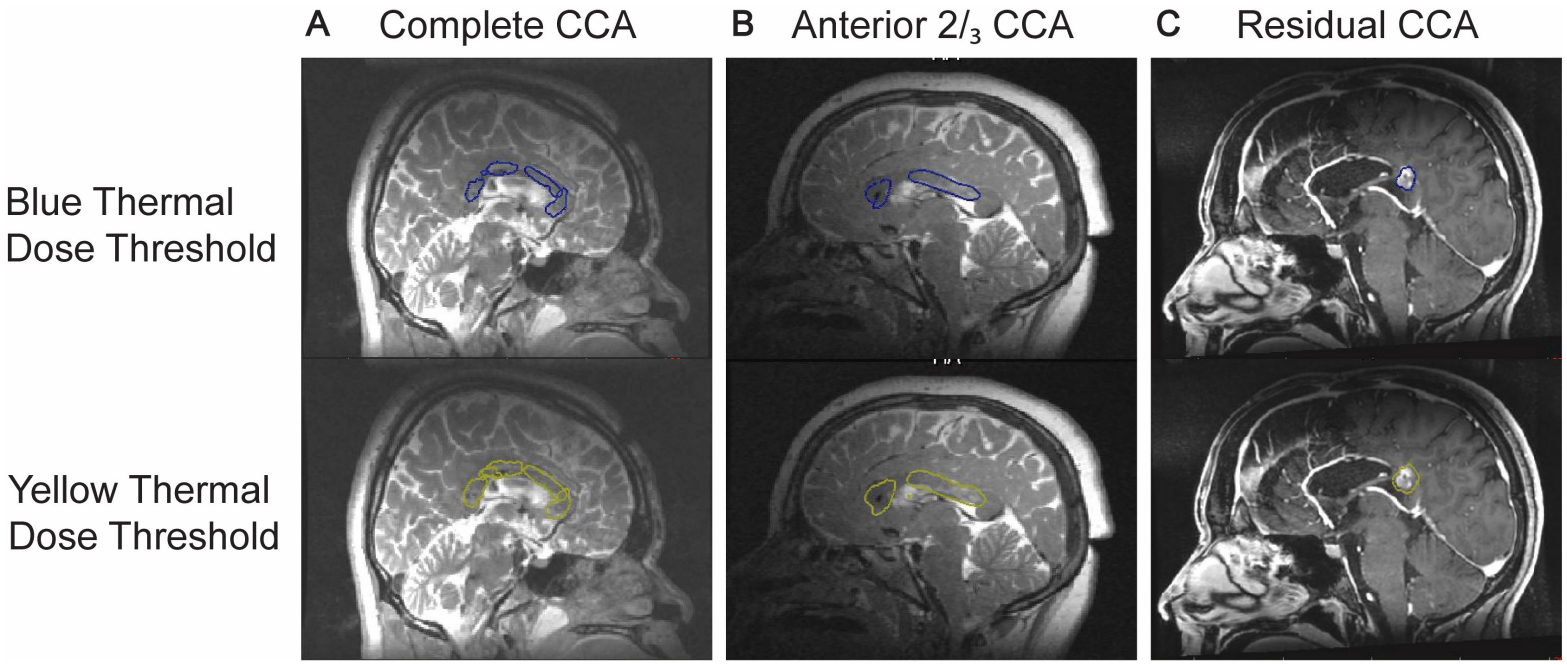
Sample images showing real time thermography when targeting the corpus callosum for **(A)** complete CCA, **(B)** anterior 2/3rds CCA, and **(C)** residual CCA.

In general, complications can potentially be avoided using MR guidance (Figure 3) and stereotactic planning with preoperative CT angiography merged with T1-weighted MRI (31, 74). Furthermore, to maximize targeting accuracy Ball et al. suggests minimizing the time interval between the removal of the titanium mandrel and reinsertion of the ablating probe; surgeons should be careful not to damage to the probe, though, because it could lead to insufficient cooling and cause overheating of the surrounding brain tissue (25). Also, given that the probes are relatively blunt, inserting them into the corpus callosum in a more perpendicular fashion—rather than a shallow angle— can reduce the of probe deflection due to the density of white matter tracts.

Neurological deficits can occur in patients undergoing CCA. Hect et al. found that 18.82% of patients experienced transient neurological deficits and 4.71% experienced permanent deficits (42). The most common transient deficits were transient hemiparesis, often occurring without damage to the supplemental motor area, and disconnection syndrome. Potential causes of supplemental motor area -like syndromes post-CCA include probe malpositioning leading to off target ablation or hemorrhage causing cingulate gyrus edema (75). Other temporary deficits reported include truncal ataxia and imbalance with one patient experiencing permanent dysarthria after complete CCA (31, 34, 37).

### 3.3 Optimizing patient selection

The major indications for CCA are similar to that of open callosotomy. Institutions consistently required evaluation by a multidisciplinary team, diagnosis with drug resistant epilepsy (evidenced by failure of two or more antiepileptics) with documentation of drop seizures, electrophysiological testing, and a neuropsychological evaluation to decide on appropriateness and extent of CCA. As noted by Aum et al. and others, LITT was especially useful for CCA when applied for patients with multiple medical comorbidities, higher risks associated with surgery, severe or complex epilepsy for which the potential benefit of callosotomy was less clear, or those who wished to avoid open operations (54).

Two major considerations for CCA candidacy are the shape of the corpus callosum and the number of trajectories needed for complete ablation (26). Thin corpus callosums pose a challenge due to their small size: the heat sink properties of cerebrospinal fluid (CSF) can result in less effective ablation. Best et al. recommends against attempting ablations in a corpus callosum that is “not much thicker than the laser catheter being used” (26). Additionally, achieving complete ablation may be challenging in patients with tortuous corpus callosums, requiring multiple trajectories and increasing the risk of complications from probe malpositioning. However, it is important to note that because the lateral corpus callosum “fans out” and becomes wider (76), a functional disconnection may still be safely achieved in some patients even with a thin corpus callosum. Other considerations for CCA candidacy include the absence of implants that could interfere with laser fiber placement (e.g., MRI-incompatible vagus nerve stimulators) and the ability to obtain an MRI scan under general anesthesia (54).

### 3.4 Limitations in the literature

The literature on CCA is limited by the predominance of observational studies and significant heterogeneity across patient populations. Variations in the extent of CCA, history of other surgeries or concurrent neuromodulation, types and etiology of epilepsy, pharmacologic management, methods for reporting seizure outcomes, and age at CCA limit the generalization of results (42).

## 4 Application of MRgLITT to hemispherotomy

### 4.1 Completion of prior disconnective surgery for refractory epilepsy

Residual connections after functional hemispherotomy is one of the most common reasons for requiring reoperation (77–80). Reoperation of failed disconnective surgeries is necessary to achieve seizure control but presents significant challenges and risks, including hydrocephalus, stroke, and infection (81–84). In 2023, Candela-Cantó et al. published a case series in which LITT was used to complete disconnective surgery post-hemispherectomy (four patients) or post- temporal occipital parietal disconnection (two patients) to address persistent or recurring seizures or to facilitate antiepileptic drug withdrawal (24). Four of the six patients were seizure free after the initial LITT operation; the remaining two patients (one temporal occipital parietal disconnection and one hemispherotomy patient) required reoperation. No complications were reported in any of these cases.

Similarly, Ravindra et al. published a single-center case series in 2023 involving five pediatric patients who underwent LITT for completion of hemispherotomy (85). These patients exhibited recurrent or persistent seizures, which were believed to result from incomplete disconnection. The mean daily seizure frequencies were as follows: 11.25 ± 5.2 before the first open surgery, 8.6 ± 9.5 after open surgery, and 1.03 ± 1.98 after LITT completion. Four out of five patients showed improvements in neuropsychiatric functioning and speech performance. Notably, none of the patients required shunt placement for hydrocephalus, which is often necessary in open reoperations.

These studies indicate that LITT is a safe and effective method for completing previous disconnective surgeries when dealing with persistent seizure semiologies. The deep-seated and small nature of persistent connections makes them particularly amenable to LITT. However, a higher amount of energy is required to ablate residual fibers: this is likely due to the cooling effect of the surrounding CSF (24).

### 4.2 Complete functional hemispherotomy

Chua et al. and Mendoza-Elias et al. reported on the use of LITT for performing functional hemispherotomies in three patients (86, 87).

Chua et al. described a 5-year-old patient with medically refractory hemiclonic seizures following a hemispheric infarction. Due to multiple comorbidities, including congenital heart disease and end-stage renal failure, open craniotomy was deemed too risky. The surgery was performed pre-transplant to avoid complications related to posttransplant immunosuppression. The patient experienced transient increased intracranial pressure and vomiting, managed with CSF drainage and dexamethasone. Postoperatively, there was an expected worsening of left hemiparesis, but the patient regained ambulation and was discharged to rehabilitation 16 days postop with no documented seizures. Within the first 5 months postoperatively she had three episodes of possible seizures at home but was seizure free at 16 months follow-up with continued moderate levetiracetam and oxcarbazepine therapy.

Chua et al. noted that the patient was an ideal candidate due to prior stroke-induced encephalomalacia of the insula which caused subinsular disconnection and generalized hemispheric volume loss, which facilitated near-complete cortical and subcortical disconnections. Despite the inability to complete tractography postoperatively, the patient's favorable seizure outcome indicated successful functional disconnection.

Mendoza et al. reported on two 11-year-old patients with epilepsy secondary to perinatal stroke, who underwent LITT hemispherotomy individually tailored using preoperative tractography. Both patients experienced postoperative hemorrhage without permanent neurological damage and were seizure-free at 9 months postoperatively, with significant improvements in quality of life. Postoperative tractography revealed preserved basal frontal and callosal streamlines. Seizure freedom despite residuals might be attributed to fake streamlines or the non-involvement of residual regions in the preoperative epileptogenic zone.

These findings suggest that LITT functional hemispherotomy could be a viable minimally invasive alternative to open procedures, potentially reducing complication rates and making the surgery more accessible. However, it may be more difficult to achieve the same level of complete 3D disconnection as in open surgery.

### 4.3 Limitations in the literature

The application of LITT in hemispherotomies is still underexplored, with existing studies primarily comprising case series. Therefore, the results cannot be generalized. Further research, including larger, more diverse patient populations, is necessary to validate these findings.

## 5 Confirming ablation of target tracts

Various methods have been employed to verify adequate ablation following application of LITT for disconnective surgeries and to explore the relationship between postoperative changes in connectivity. These methods include MRI scans performed intraoperatively (25), immediate confirmation postoperatively using T1-weighted or FLAIR images (88), MRI scans obtained more than 3 months after the LITT procedure (26), and diffusion-weighted imaging (28). Additionally, multimodal approaches integrating electrophysiology-based methods have been utilized (30). Despite the diversity in approaches, there continues to be loose, inconsistent correlations between extent of ablation and patient outcomes.

## 6 Discussion

The literature supports the safe and effective application of LITT to disconnective epilepsy surgeries. This shift toward minimally invasive approaches potentially increases accessibility for patients and families hesitant to undergo traditional open brain surgeries. Unfortunately, the existing literature primarily consists of observational studies, characterized by considerable variation in patient populations, methodologies for assessing patient outcomes, and criteria for assessing surgical success. This complicates generalizability. Moving forward there is a critical need for multi-center, large-scale studies with uniform protocols for patient selection and outcome measurement. Such efforts are essential to address these gaps and provide robust evidence to guide clinical practice effectively.

In addition to the need for improved studies, there is a need for further investigation into the role of VNS and seizure control. VNS devices are being implanted before, simultaneously with, and after performing CCA (42) which complicates our ability to understand the impact of either procedure on seizure control. For example, Ordaz et al. reported that five out of 11 patients had VNS before undergoing CCA procedure (22). Among these patients, four experienced a 100% decrease in drop seizures while one had no decrease in drop seizures. This positive effect of preoperative VNS was not seen as strongly in the open corpus callosotomy cohort. Paired with the all-or-nothing response to CCA, this suggests that there are both patient-specific and intervention-specific factors contributing to seizure control but with the current data available we are unable to identify them.

A better understanding of how patient characteristics and intervention modalities synergistically improve seizure outcomes can significantly enhance both surgical decision-making and patient comfort. The high prevalence of dual VNS and CCA interventions, coupled with unclear guidelines for selecting patients who would actually benefit from both, may lead to unnecessary procedures and increased time away from school and work. Furthermore, it is important to consider the financial impact of these surgical redundancies, especially if VNS is being performed in patients that would have otherwise achieved total drop seizure freedom with CCA alone. This high prevalence of dual procedures may be due to the relative novelty LITT as it was first applied to CCA in 2016. Over time, as CCA becomes more widely available, there may be a downwards trend in prior VNS.

Another significant gap in the literature is an insufficient understanding of the structural components to epileptogenic networks and how macro- and microstructural changes in the corpus callosum affect said network and, subsequently, patient outcomes. Various methods for assessing extent of ablation have been employed. Best et al. used measurements from T1 postcontrast and diffusion-weighted imaging (26), Caruso et al. used MRI scans (23), and Ordaz et al. used free tracing of T1 weighted images (22). While all authors found that there were high percentages of ablation, and therefore some degree of correlation between ablation and seizure freedom, the correlation was not strong, and the sample sizes were too small to perform rigorous statistical analyses. These findings suggests that the percent of disconnection alone may not be a specific enough measure to predict seizure freedom. Therefore, more systematic investigations into patient-specific macro-and microstructural anatomy are necessary to better understand structural connectivity within the epileptogenic network. Such research could illuminate how surgical interventions succeed or fail in disrupting said networks and contribute to seizure freedom.
